# Optimizing action separation and integration through moderate practice

**DOI:** 10.3758/s13423-025-02782-7

**Published:** 2026-02-19

**Authors:** Jens Kürten, Julia Escher, Tim Raettig, Lynn Huestegge

**Affiliations:** Department of Psychology, University of Würzburg, Röntgenring 11, 97070 Würzburg, Bavaria Germany

**Keywords:** Multiple action control, Inhibitory control, Eye movements, Action representation, Dual-action practice

## Abstract

Coordinating multiple actions simultaneously often results in performance costs. However, sometimes dual actions can also be easier to perform than single actions, in particular when inhibitory control is needed during single-action control. For example, an erroneous co-execution of another response or slower single- (vs. dual-) action execution may be observed. In two experiments with different extents of practice (six blocks in Experiment 1, 12 blocks in Experiment 2), we looked into two potential practice-related sources of such effects: increased action coupling with time-on-task versus initial crosstalk followed by improved separation of action representations. Participants executed single left/right saccadic eye movements, uttered single “left”/ “right” directional words, or performed both actions simultaneously based on prior cues. Results showed high saccadic co-execution error rates and vocal dual-action benefits in response times at the beginning, with a significant reduction over the course of the session. The findings support the assumption of a more pronounced separation of action representations with practice, while at the same time improvements in temporal dual-action coordination took place. The study thus highlights the optimization of action representations resulting from moderate practice.

**Supplementary Information:**

The online version contains supplementary material available at 10.3758/s13423-025-02782-7.

## Introduction

Many studies have consistently demonstrated difficulties in coordinating two actions at the same time compared to performing only one action (dual-action *costs*; see Koch et al., 2018, for a review). However, recent research has pointed towards the possibility of an opposite effect: performing two highly code-compatible actions can also be easier than performing only one action alone (dual-action *benefit*; see Huestegge & Koch, 2014). Corresponding studies typically involve frequent switches between dual-action and single-action execution on a trial-by-trial basis. This has led to frequent erroneous co-execution of the unwarranted action when only one action is required, resulting in overall worse performance in single-action conditions. Here, we investigated how this bias is shaped by increasing time on task. Specifically, we tried to discern whether the tendency towards erroneous co-execution mainly stems from (a) a stronger coupling of actions with increasing dual-response practice, so that co-execution errors should become more frequent with time-on-task (i.e., “from separation to integration”), or (b) early crosstalk between task components, with a more pronounced separation (or shielding; see e.g., Fischer et al., 2014) and thus fewer co-execution errors over time (i.e., “from integration to separation”).

Co-execution errors in single-action control and the resulting dual-action benefits have been attributed to crosstalk between overlapping, highly compatible response-relevant codes, which produces a spillover of activation into an unwarranted yet pre-potent effector system during response selection (Huestegge & Koch, 2010; see also Hommel, 2020). Under such conditions, performing a spatially compatible dual action may even become the default response (Raettig & Huestegge, 2021, 2023). Co-execution errors can therefore be conceptualized as failures of selective inhibitory control over the non-required effector system, which is necessary for successful single-action selection (see Wadsley et al., 2022, for a review). An important yet unresolved question concerns whether and how co-execution tendencies, and thus dual-action benefits, change with (moderate) practice.

Research on practice-related changes in the processes and representations underlying human (multiple) action control has a long tradition (e.g., Logan, 1988; Schneider & Shiffrin, 1977; for reviews, see Koch et al., 2018; von Bastian et al., 2022). Within the domain of multiple-action control, practice effects are of particular interest because they inform whether performance limitations reflect structural constraints or instead arise from strategic and representational factors (e.g., Karlin & Kestenbaum, 1968; Van Selst et al., 1999). Prior work has focused almost exclusively on the reduction of dual-action (or dual-task) costs with practice, and theoretical explanations have accordingly centered on this performance improvement. Such reductions have been attributed to the automatization of individual response processing or to stronger integration of two responses into a single, unitary representation (Ruthruff et al., 2006). More recently, these improvements have also been linked to enhanced executive coordination skills and to more efficient instantiation of task representations in working memory (Schubert et al., 2024; Strobach et al., 2014). By contrast, the implications of practice for dual-action benefits as indexed by co-execution errors in single-action trials have received no attention to date. The present study addresses this gap, thereby extending the discussion of practice-related changes in multiple-action control to contexts where interference is strongest in single-action trials due to the tendency to co-activate additional responses.

In the present study, participants responded with left/right saccadic eye movements and/or by uttering directional words (“left”/“right”) to arbitrary shape stimuli (circle or diamond) across six blocks (Experiment 1) or 12 blocks (Experiment 2) of practice. Trial type (single-saccade, single-vocal, or dual) was cued by the color of a preceding fixation cross in Experiment 1 and by the color of the shape itself in Experiment 2, whereas the shape determined response direction. Shape-to-direction mappings were spatially compatible for both effectors, resulting in strong response compatibility (Hazeltine, 2005). Prior research with a saccade-manual effector-system combination has consistently shown a predominance of saccadic co-execution errors in single-manual trials and comparatively fewer manual co-execution errors in single-saccade trials (Kürten et al., 2023a, 2023b, 2024). If the general mechanism underlying dual-action benefits transfers to a saccade-vocal effector-system combination, we would therefore also expect frequent false-positive saccades in single-vocal trials. By contrast, overt vocal co-execution errors in single-saccade trials should be relatively rare (Raettig & Huestegge, 2018, 2021; see also Raettig & Huestegge, 2023), as speech articulation may have a higher execution threshold than saccadic eye movements, which are executed frequently and often quasi-reflexively to support visual perception (Huestegge et al., 2019; Munoz & Everling, 2004).

The key condition of interest was therefore the single-vocal condition, in which we measured saccadic co-execution errors and the response times (RTs) of correct vocal responses. Single-vocal RTs were also compared to those in dual-action trials to assess dual-action costs or benefits as an additional indicator of action integration and separation. If action representations shift from separation to integration, we would expect co-execution errors to increase over time, accompanied by stronger practice effects in dual-action than in single-action RTs. This pattern would be consistent with the idea that sufficient practice can merge two tasks (or actions) into one, thereby reducing dual-task (or dual-action) costs (Hazeltine et al., 2007; Strobach, 2020). Conversely, a shift from integration to separation would predict an initially high co-execution bias that diminishes with practice, along with relatively stronger gains in single-action RTs, potentially due to automatization of individual responses. This view aligns with accounts suggesting that new tasks are initially represented in a “blended” form that facilitates flexibility but at the same time may increase crosstalk (Musslick & Cohen, 2021; Musslick et al., 2020). With practice, task-relevant response representations may then become more distinct, for example, by segregating the task set according to effector systems and by improving shielding of these task boundaries (Dreisbach & Haider, 2008; Fischer et al., 2014; Philipp & Koch, 2011). This separation might even lead to measurable serialization of response execution in dual-action trials, reflected by increasing inter-response intervals with time-on-task (Logan & Gordon, 2001). Finally, a hybrid account predicts optimization of both separation and integration: Practice should reduce co-execution errors and speed up single-action responses while also improving dual-action performance, in line with the idea of a more efficient instantiation of task representations in working memory with ongoing practice (Schubert et al., 2024). This pattern may further reflect the formation of an integrated, holistic dual-action representation that is distinct from either single action (Huestegge et al., 2023).

## Methods

Experiment 1 was originally conducted as a pilot study, and the specific hypotheses tested here were not preregistered. Experiment 2 was conducted to replicate and extend the findings of Experiment 1 and the preregistration can be obtained from: 10.17605/OSF.IO/YGF3M.

### Sample

Thirty participants took part in Experiment 1, and 36 took part in Experiment 2. We excluded three datasets from Experiment 1 and eight datasets from Experiment 2 due to exceedingly high overall error rates (> 30%) or technical failures of the voice key in all blocks.^1^ The final sample of Experiment 1 thus comprised 27 participants (mean age = 23.1 years, *SD* = 3.3; 81.48% female, 92.59% right-handed), and data from 28 participants (mean age = 25.5 years, *SD* = 3.7, 75% female, 92.86% right-handed) were analyzed in Experiment 2. We conducted an a priori simulation-based power analysis (via the *simr* R package, v1.0.7) based on the lower bound of the 60% confidence interval of the main effect of block on saccade co-execution errors in Experiment 1 to plan the sample size for Experiment 2, accounting for a potential overestimation of the effect (Perugini et al., 2014). The analysis indicated that five participants would suffice to detect a main effect of the block factor on the probability of saccade co-execution errors with a power of 1 − $\beta$  = 80% at a significance level of $\alpha$ = 5%. We decided to collect data from more participants to increase comparability with Experiment 1 and to potentially detect differences between individual blocks. All participants had normal or corrected-to-normal vision, gave informed consent, and were compensated monetarily or with partial course credit.

### Materials and task

Participants were seated in front of a computer screen (Experiment 1: 21-in. CRT, 100 Hz, 1,024 × 768 px, 67-cm viewing distance; Experiment 2: 25-in. Fast IPS Dell Alienware AW2523HF, 360 Hz, 1,920 × 1,080 px, 70-cm viewing distance). Both experiments were programmed using PsychoPy (Experiment 1: v2022.2.5, Experiment 2: v2024.2.4; Peirce et al., 2019). Right-eye saccade RTs, directions, and amplitudes were measured with an EyeLink 1000 (SR Research, Ontario, Canada) at 1,000 Hz, with head movements minimized by a chinrest. Vocal responses were captured using a Sennheiser E835-S microphone. Vocal RTs were determined by PsychoPy’s voice-key routine, and vocal response identity (for error coding) was recorded manually by the experimenter. Stimuli consisted of a central white or colored (red, yellow, blue) plus sign (0.33° VA) serving as a fixation cross, a central white or colored shape (circle or diamond; 0.67° VA), and two black rectangles (0.33° VA, 1-pixel white border) as saccade landing targets presented to the left and right of the screen center at an eccentricity of 8° VA (Experiment 1) or 10° VA (Experiment 2). All stimuli appeared on a black background. After written and verbal instructions emphasizing both speed and accuracy, participants completed eye-tracker calibration and a 30-trial practice block (not analyzed). The main task comprised six (Experiment 1) or 12 (Experiment 2) blocks of 60 randomly ordered trials, each block preceded by a drift check and, if needed, recalibration of the eye-tracker. In Experiment 1, trials began with the display of the fixation cross and landing targets. After 250 ms, the cross changed color to indicate trial type (e.g., red: single saccade, yellow: single vocal, blue: dual; color-to-trial type mapping counterbalanced across participants), and after another 250 ms, a white shape replaced the cross to indicate response direction (e.g., circle: left, diamond: right; shape-to-direction mapping counterbalanced across participants). Stimuli remained visible for 2,000 ms, followed by a 500 ms blank inter-trial interval (ITI). In Experiment 2, the fixation cross (500 ms) was followed by a colored shape (max. 2,000 ms) that simultaneously conveyed trial type (via its color) and response direction (via its shape), thereby eliminating any opportunity for proactive control of effector systems. The ITI remained constant at 500 ms.

### Data analysis

All analyses were conducted in R (version 4.5.0, R CoreTeam, 2025), the *papaja* package (v0.1.3) was used to facilitate reproducible reporting (Aust & Barth, 2022). Valid saccade responses were defined as landing at least half-way (Experiment 1: 4° VA, Experiment 2: 5° VA) towards one of the two landing targets. Valid vocal responses were defined as vocalizations of directional words associated with a recorded vocal RT. Other sounds triggering the voice key (e.g., coughs) were considered invalid. From all analyses, we excluded invalid trials (4% of trials in Experiment 1; 5.89% in Experiment 2) and premature responses (saccade RTs < 50 ms, vocal RTs < 150 ms, 0.03% of trials in Experiment 1; 0.11% in Experiment 2). The remaining trials were analyzed for co-execution errors, that is false-positive execution of a saccade/vocal response when not required in single-action trials.^2^ From RT analyses, we further excluded all types of response error (8.18% of trials in Experiment 1; 14.08% in Experiment 2) and outliers (RTs >  ± 3 *SD*s away from cell mean, 0.97% of trials in Experiment 1; 0.52% in Experiment 1). Excluding error trials from RT analyses resulted in the factor trial type comprising only two levels in each response modality: single saccade and dual in saccades, and single vocal and dual in vocal responses.

We analyzed the probability of false-positive saccade errors in single-vocal trials (co-execution errors) separately for each experiment using generalized linear mixed models (GLMMs) with block (levels one through six in Experiment 1, levels one through 12 in Experiment 2) as a within-subject fixed effect and participant as a grouping variable. We analyzed RTs of correct vocal responses in single-vocal and dual-action trials separately for each experiment using linear mixed models (LMMs) with block and trial type (single vocal vs. dual action) as within-subject fixed effects and participant as a grouping variable. We aimed for the maximal random-effects structure supported by the data following the recommendations by Barr et al., (2013; see also Singmann & Kellen, 2019). For GLMMs (co-execution errors), *p* values for fixed effects were obtained using log-likelihood ratio tests. For LMMs (RTs), we used Satterthwaite approximations for Type III tests. Significant main effects with more than two factor levels and significant interactions were decomposed using Bonferroni-adjusted post hoc comparisons of the models’ estimated marginal means (EMMs, ms for RTs, probability for co-execution errors). We used asymptotic degrees of freedom for all follow-up comparisons as implemented in the *emmeans* package (v1.11.2) in R.

## Results and discussion

### Saccade Co-execution Errors

**Fig. 1:**
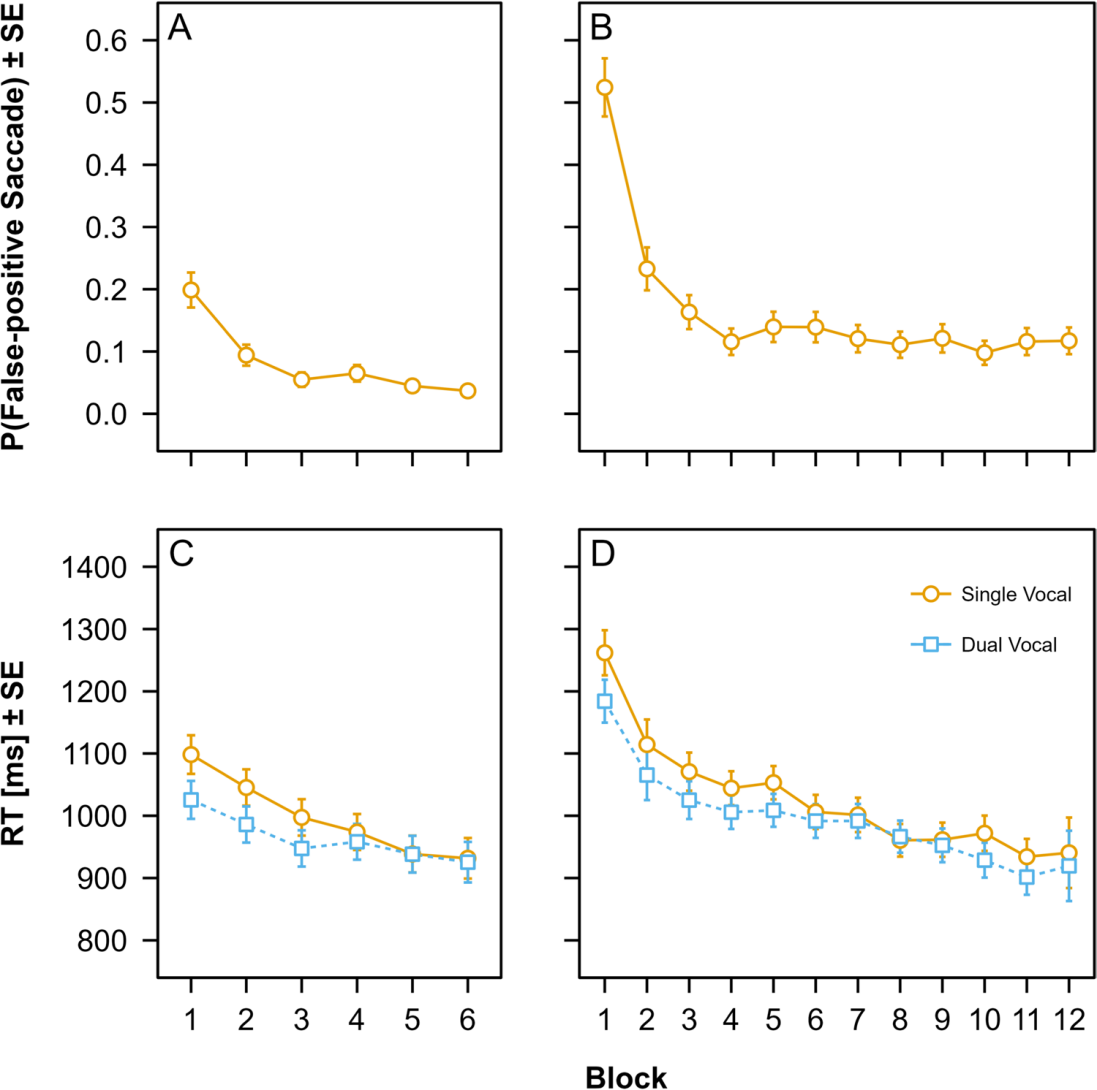
Main results of Experiments 1 and 2. Data points represent estimated marginal means (EMMs) predicted by the (generalized) linear mixed models fit to the probability of saccade co-execution errors in single-vocal trials in Experiment 1 (**A**) and Experiment 2 (**B**), and vocal response times (RTs) in single-vocal and dual-action trials in Experiment 1 (**C**) and Experiment 2 (**D**), respectively. Error bars represent model-based standard errors of the EMMs. See the online article for the color version of this figure

The probability of committing false-positive saccades decreased significantly with time-on-task both in Experiment 1 (Fig. 1 A), ${\chi }^{2}\left(5\right)=106.26$, $p<.001$, and in Experiment 2 (Fig. 1 B), ${\chi }^{2}\left(11\right)=397.54$, $p<.001$. In Experiment 1, we found a sharp reduction in saccade co-execution error probability from Block 1 ($p=0.20$) to Block 2 ($p=0.09$, ${p}_{\mathrm{Bonferroni}\left(5\right)}<.001$). Subsequent between-block changes were non-significant (all ${ps}_{\mathrm{Bonferroni}\left(5\right)}\ge .053$). In Experiment 2, we observed a similar pattern. False-positive saccade probability dropped substantially from Block 1 ($p=0.52$) to Block 2 ($p=0.23$, ${p}_{\mathrm{Bonferroni}\left(11\right)}<.001$), and then leveled off with non-significant differences between subsequent blocks (all ${ps}_{\mathrm{Bonferroni}\left(11\right)}\ge .056$

### Vocal RTs

In Experiment 1 (Fig. 1 C), we found no significant main effect of trial type on vocal RTs, $F\left(1,26.16\right)=3.35$, $p=.079$. Overall, vocal RTs decreased significantly as a function of time-on-task, $F\left(5,41.83\right)=15.69$, $p<.001$. Importantly, there was a significant interaction between trial type and time-on-task, $F\left(5,5450.55\right)=4.29$, $p<.001$. In Block 1 participants showed significant dual-action benefits (${p}_{\mathrm{Bonferroni}\left(6\right)}=.011$). In Blocks 2 and 3, numerical dual-action benefits were non-significant (both ${ps}_{\mathrm{Bonferroni}\left(6\right)}\ge .053$). In Blocks 4 through 6, there were minimal differences between single-action and dual-action RTs (all ${ps}_{\mathrm{Bonferroni}\left(6\right)}\ge .053$). In Experiment 2 (Fig. 1 D), vocal responses were overall faster in dual-action trials ($M=995$ ms) compared to single-action trials ($M=1,027$ ms), $F\left(1,27.43\right)=5.74$, $p=.024$. Generally, vocal RTs decreased as a function of time-on-task, $F\left(11,47.18\right)=17.55$, $p<.001$. Importantly, there was again a significant interaction between trial type and time-on-task, $F\left(11,10250.78\right)=2.22$, $p=.011$. Significant dual-action benefits in Block 1 (${p}_{\mathrm{Bonferroni}\left(12\right)}=.006$) were reduced, resulting in non-significant numerical benefits in Blocks 2 through 7 (all ${ps}_{\mathrm{Bonferroni}\left(12\right)}\ge .132$), with a tendency towards non-significant dual-action *costs* in Block 8 (${p}_{\mathrm{Bonferroni}\left(12\right)}>.999$). In Blocks 9 through 12, non-significant numerical dual-action benefits re-emerged (all ${ps}_{\mathrm{Bonferroni}\left(12\right)}\ge .262$).

## General discussion

We aimed to investigate the effects of practice with increasing time-on-task on action integration and separation in multiple-action control. Specifically, we focused on situations requiring a single (here: vocal) response in which another, highly compatible action (here: a spatially aligned saccade) is sometimes inadvertently co-executed. Participants alternated between performing single directional (left/right) saccades, single vocal utterances of directional words (“left”/“right”), or both together in a spatially compatible manner over six blocks of practice in Experiment 1 and 12 blocks of practice in Experiment 2. Performance in single-vocal trials was of primary interest. In these trials, the rate of false-positive saccades (co-execution errors) and correct vocal RTs (compared with vocal RTs in dual-action trials) served as indicators of a failure to separate the representations of both effector systems.

Overall, saccade co-execution errors were more frequent and vocal responses slower in Experiment 2 than in Experiment 1, presumably due to the integration of the cue (indicating trial type) and the stimulus (indicating response direction).^3^ Importantly, in both experiments, responses became more accurate and faster over the course of the experimental session. Vocal RTs showed an initial dual-action benefit early in practice, which diminished with increasing time-on-task. Crucially, this reduction in RTs was accompanied by a marked reduction in saccade co-execution error rates in single-vocal trials. The reduction was strongest after the first block of trials, with smaller (non-significant) decreases over subsequent blocks. However, false-positive saccade error rates did not drop to zero even after 12 blocks of practice in Experiment 2, indicating that the observed co-execution tendency was not entirely attributable to unfamiliarity with the task.

The reduction in saccadic co-execution error rates and the reduction of vocal dual-action benefits are clearly consistent with a representational change *from integration to separation* and at odds with a change *from separation to integration*. Participants became more proficient at separating effector systems in trials calling for an isolated engagement of effectors, likely supported by improved selective inhibition of the non-relevant effector system (Wadsley et al., 2022). However, at the same time, the present data do not suggest that the shift from integration to separation was absolute. Participants did not appear to strictly separate both effector systems, which might have been reflected by a pronounced serial response execution in dual-action trials (Logan & Gordon, 2001). Instead, we also found a practice advantage in dual-action trials even for the slower (vocal) response as well as a slight (non-significant) *reduction* (rather than increase) in the mean inter-response interval between saccades and vocal responses in dual-action trials (see Table S3 and Fig. S3, Electronic Supplementary Material), tentatively suggesting an improved synchronization rather than serialization of dual actions after practice. This could indicate a stronger segregation of effector system representations over time with a concurrent improvement in general dual-action coordination. This is well in line with accounts of practice-related reductions in dual-task *costs* that assume improved executive coordination skills (Schubert et al., 2024; Strobach, 2023) or better shielding of mental action representations over time (Musslick & Cohen, 2021). One possibility of how such a representational sharpening could play out would be that instead of separating between saccade and vocal actions alone (i.e., between single actions that need to be coordinated in dual-action trials), participants may have formed a distinct dual-action representation that is separable from either single-action representation (Huestegge et al., 2023). Based on the present data alone, it is difficult to ascertain whether participants did indeed form such a holistic dual-action representation and, if yes, when exactly this occurred. Future studies should therefore develop better empirical indicators of “integration,” for example, based on transitional RTs between trials (Dykstra et al., 2022), based on the inter-response times (Miller & Ulrich, 2008; Ulrich & Miller, 2008), or by utilizing more continuous response measures to capture the dynamics of action selection (Scherbaum et al., 2015).

The initial bias toward saccade co-execution in single-vocal trials was likely driven by the strong code compatibility between actions (e.g., left-left) to be performed with either effector (Kornblum et al., 1990). Separating responses early on might be easier, or even necessary, under incompatible task demands for saccade and vocal responses, such as when the same stimulus is associated with a leftward saccade but vocalization of the word “right,” a prediction that could be tested explicitly in future work. Another potential reason for strong crosstalk at the beginning of practice may have been the use of a single imperative stimulus to trigger both single and dual actions (based on a prior or concurrent cue), which may have promoted a parallel rather than a serial response-selection strategy and thus increased susceptibility to crosstalk (Fagot & Pashler, 1992; Fischer et al., 2014). Future studies could therefore employ distinct stimuli for each effector system, as is typically implemented in the dual-task literature (for reviews, see Koch et al., 2018; Pashler, 1994). In such a setup, single-action trials would contain a go stimulus indicating the required effector and a no-go stimulus indicating the non-required effector, which might in turn enable a clearer separation of the two response representations early on. Interestingly, there was considerable inter-individual variability in both the initial co-execution bias and its reduction with practice. This variability may reflect differences in the format of action representations maintained by individual participants (Ackerman, 1987; Hazeltine et al., 2022; Hommel & Colzato, 2017). Although the present sample is too small to reliably distinguish between such individual representational preferences, this idea warrants systematic investigation in future research.

To conclude, when participants frequently alternated between single and dual actions under conditions of high (here: spatial) code overlap between actions, there was a strong initial bias toward co-executing prepotent but unwarranted behavior in single-action trials accompanied by a dual-action RT benefit. The present results demonstrate that this bias along with the RT benefit can be reduced rapidly with only moderate practice. At the same time, dual-action execution did not show signs of impairment or increasing serialization over time. Taken together, these findings suggest an optimization of both action separation and integration rather than a universal shift from separation to integration or vice versa.

## Supplementary Information

Below is the link to the electronic supplementary material.

Supplementary file1 (DOCX 774 kb)

## Data Availability

Preregistrations, study materials, raw data, and analysis scripts are publicly available at: 10.17605/OSF.IO/EF7PC

## Footnotes

1 Due to a loose connection, the microphone failed to record vocal responses (and thus vocal RTs) in some blocks for some participants. We excluded participants with a lack of any vocal RT data entirely, but retained data from those with only occasional microphone failures, as these can be considered random and thus unlikely to severely bias the results.

2 Analyses of all error types (false-positive, false-negative, and directional) committed with either effector system in Experiment 1 and Experiment 2 (Table S1 and Fig. S1) as well as analyses of saccade RTs in Experiment 1 and Experiment 2 (Table S2 and Fig. S2) are included in the Electronic Supplementary Material.

3 Note, however, that the experiments were not entirely comparable beyond the difference in cueing procedure, so that we cannot rule out other factors influencing the overall rate of co-execution errors.
