# Supplementary material for: Optimizing action separation and integration through moderate practice
Source: Psychon Bull Rev. 2026 Feb 19;33(2):89. doi: 10.3758/s13423-025-02782-7 (PMC12920362; doi:10.3758/s13423-025-02782-7)
Supplement: Supplementary file 1 — Supplementary file1 (DOCX 774 kb) [file 13423_2025_2782_MOESM1_ESM.docx]

# Supplement to: Optimizing Action Separation and Integration Through Moderate Practice

Jens Kürten^1^, Julia Escher^2^, Tim Raettig^1^, and Lynn Huestegge^1^

^1^Department of Psychology, University of Würzburg

^2^Würzburg, Bavaria

September 29, 2025

# Author Note

Jens Kürten
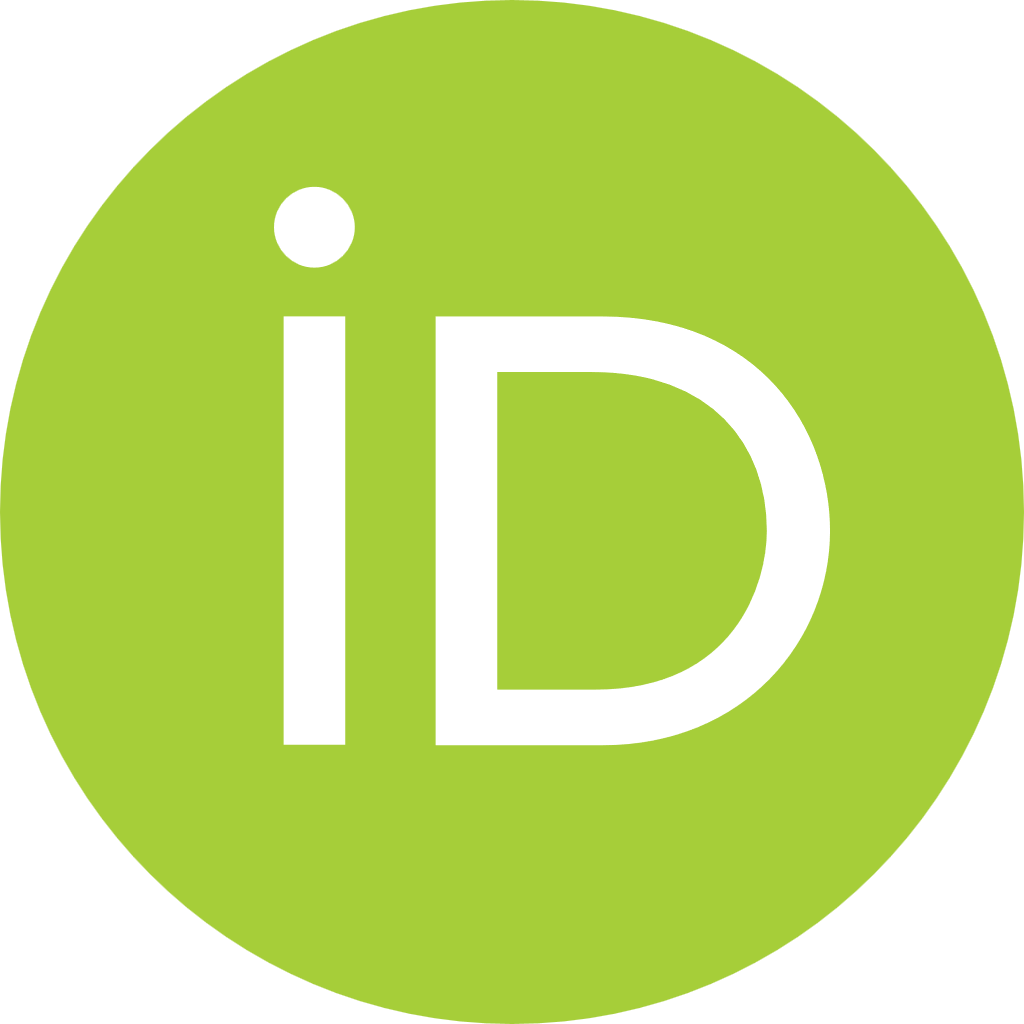
 <https://orcid.org/0000-0002-5903-1341>

Tim Raettig
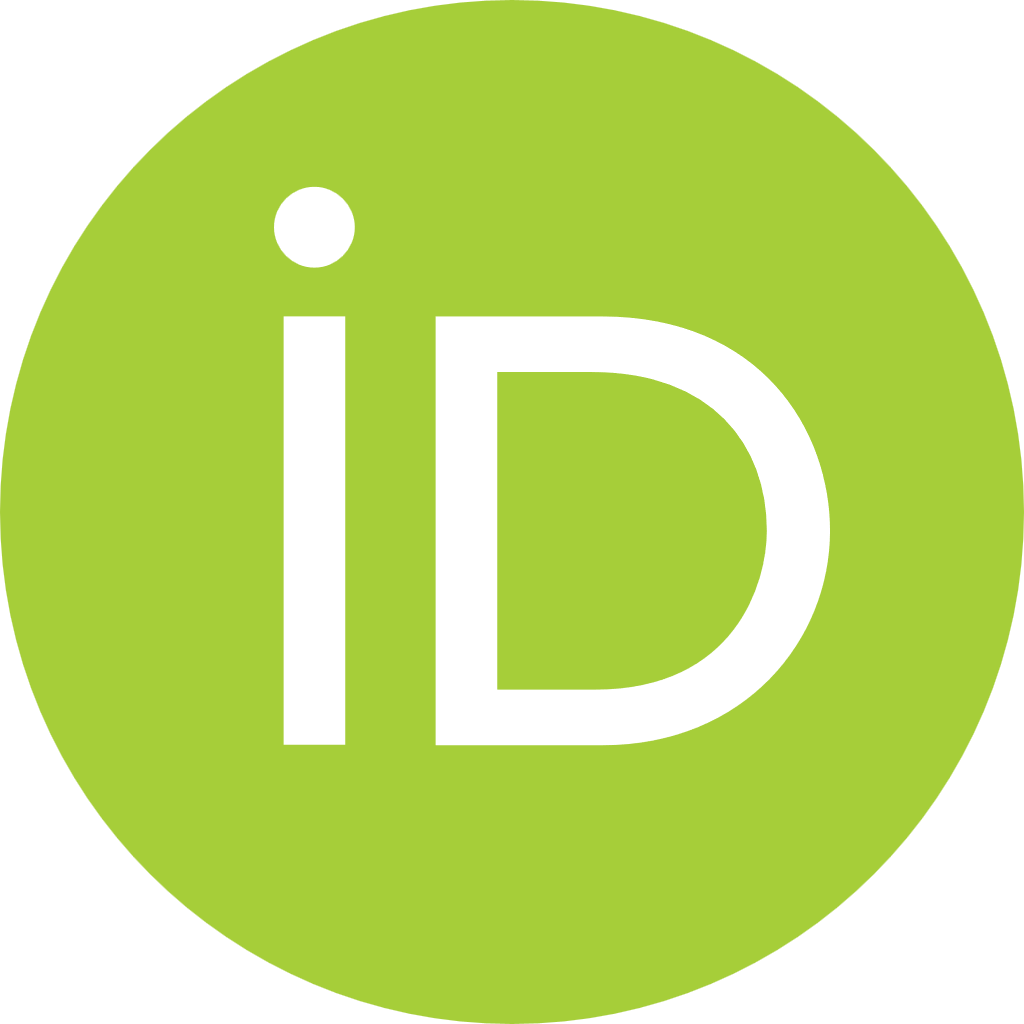
 <https://orcid.org/0000-0003-3812-2671>

Lynn Huestegge
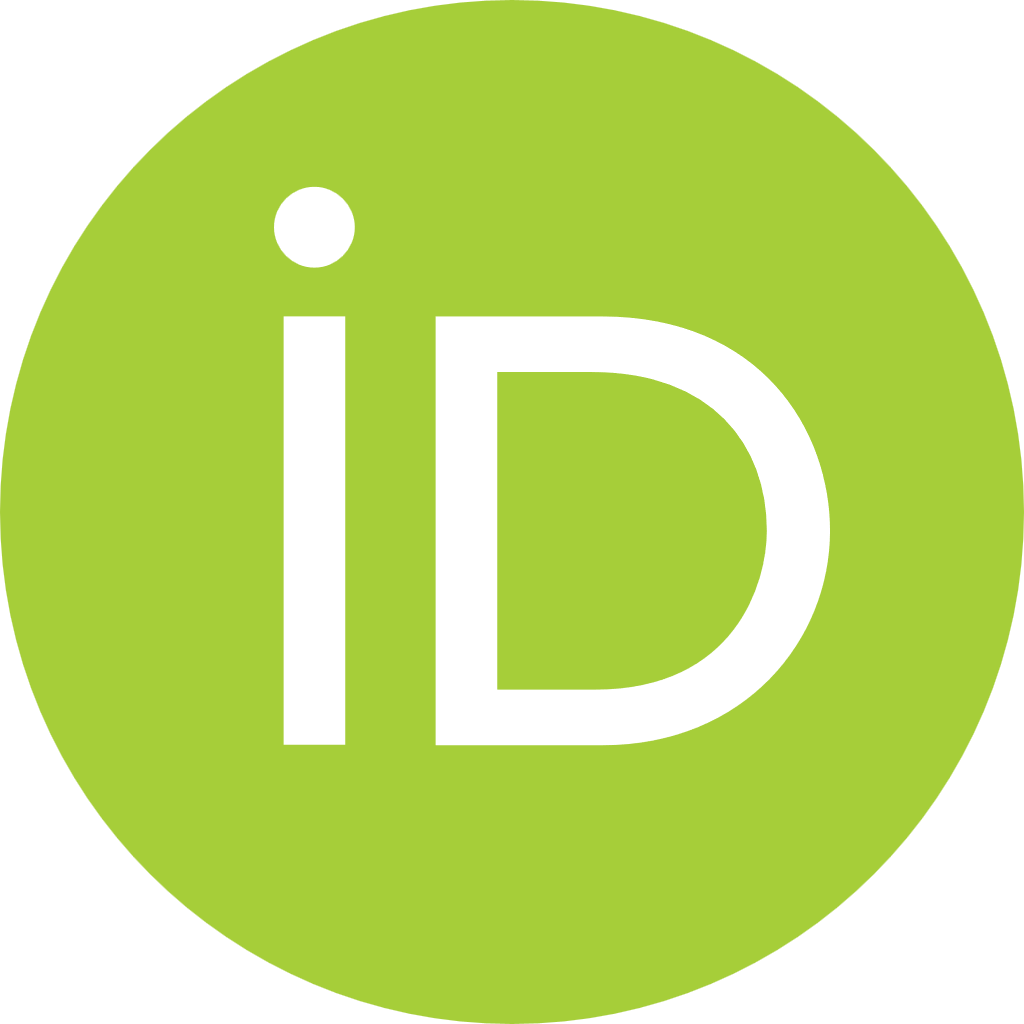
 <https://orcid.org/0000-0002-1323-7336>

Preregistrations, study materials, raw data, and analysis scripts are publicly available at https://osf.io/ef7pc/?view_only=6df77543f24d4772b4d8e5853d34f4d2 The authors have no conflicts of interest to disclose.

Author roles were classified using the Contributor Role Taxonomy (CRediT; https://credit.niso.org/) as follows: *Jens Kürten***:** conceptualization, writing – original draft (lead), formal analysis, and data curation. *Julia Escher***:** conceptualization, data curation, and writing – original draft (supporting). *Tim Raettig***:** writing – review & editing. *Lynn Huestegge***:** conceptualization, project administration, supervision, and writing – review & editing

Correspondence concerning this article should be addressed to Jens Kürten, Department of Psychology, University of Würzburg, Röntgenring 11, Würzburg, Bavaria 97070, Germany, Email: [jens.kuerten@uni-wuerzburg.de](mailto:jens.kuerten@uni-wuerzburg.de)

# Abstract

Coordinating multiple actions simultaneously often results in performance costs. However, sometimes dual actions can also be easier to perform than single actions, in particular when inhibitory control is needed during single-action control. For example, an erroneous co-execution of another response or slower single- (vs. dual-) action execution may be observed. In two experiments with differential extent of practice (6 blocks in Experiment 1, 12 blocks in Experiment 2), we looked into two potential practice-related sources of such effects: increased action coupling with time-on-task vs. initial crosstalk followed by improved action separation. Participants executed single left/right saccadic eye movements, uttered single “left”/”right” directional words, or performed both actions simultaneously based on prior cues. Results showed high saccadic co-execution error rates and vocal dual-action benefits in response times at the beginning, with a significant reduction over the course of the session. The findings support the assumption of a more pronounced separation of action representations with practice, while, at the same time improvements in temporal dual-action coordination took place. The study thus highlights the optimization of action representations resulting from moderate practice by balancing effector system separation and improved temporal dual-action coordination.

*Keywords*: multiple action control, inhibitory control, eye movements, action representation, dual-action practice

*Word Count*: 67

# Supplement to: Optimizing Action Separation and Integration Through Moderate Practice

# Additional Results

## Error Types

Table S1

Results of GLMMs Fitted to Error Types

| Experiment | Modality | Effect | $\chi^{2}$ | $df$ | $p$ |
| --- | --- | --- | --- | --- | --- |
| 1 | Vocal | Block | 32.70 | 5 | < .001 |
| 1 | Vocal | Error Type | 1.84 | 2 | .399 |
| 1 | Vocal | Block $\times$ Error Type | 17.90 | 10 | .057 |
| 1 | Saccade | Block | 39.57 | 5 | < .001 |
| 1 | Saccade | Error Type | 117.97 | 2 | < .001 |
| 1 | Saccade | Block $\times$ Error Type | 18.19 | 10 | .052 |
| 2 | Vocal | Block | 71.64 | 11 | < .001 |
| 2 | Vocal | Error Type | 9.15 | 2 | .010 |
| 2 | Vocal | Block $\times$ Error Type | 129.13 | 22 | < .001 |
| 2 | Saccade | Block | 97.00 | 11 | < .001 |
| 2 | Saccade | Error Type | 106.30 | 2 | < .001 |
| 2 | Saccade | Block $\times$ Error Type | 42.30 | 22 | .006 |

*Note*. Wald χ² tests of fixed effects from generalized linear mixed models predicting the probability of an error as a function of time-on-task (Block) and Error Type. Separate models were estimated for the vocal and saccade modalities in Experiments 1 and 2. All models used a logit link and included random intercepts for participants (with random slopes where justified). Degrees of freedom (df) refer to the χ² distribution.

Figure S1

Estimated Marginal Means of the Probability of Different Types of Error


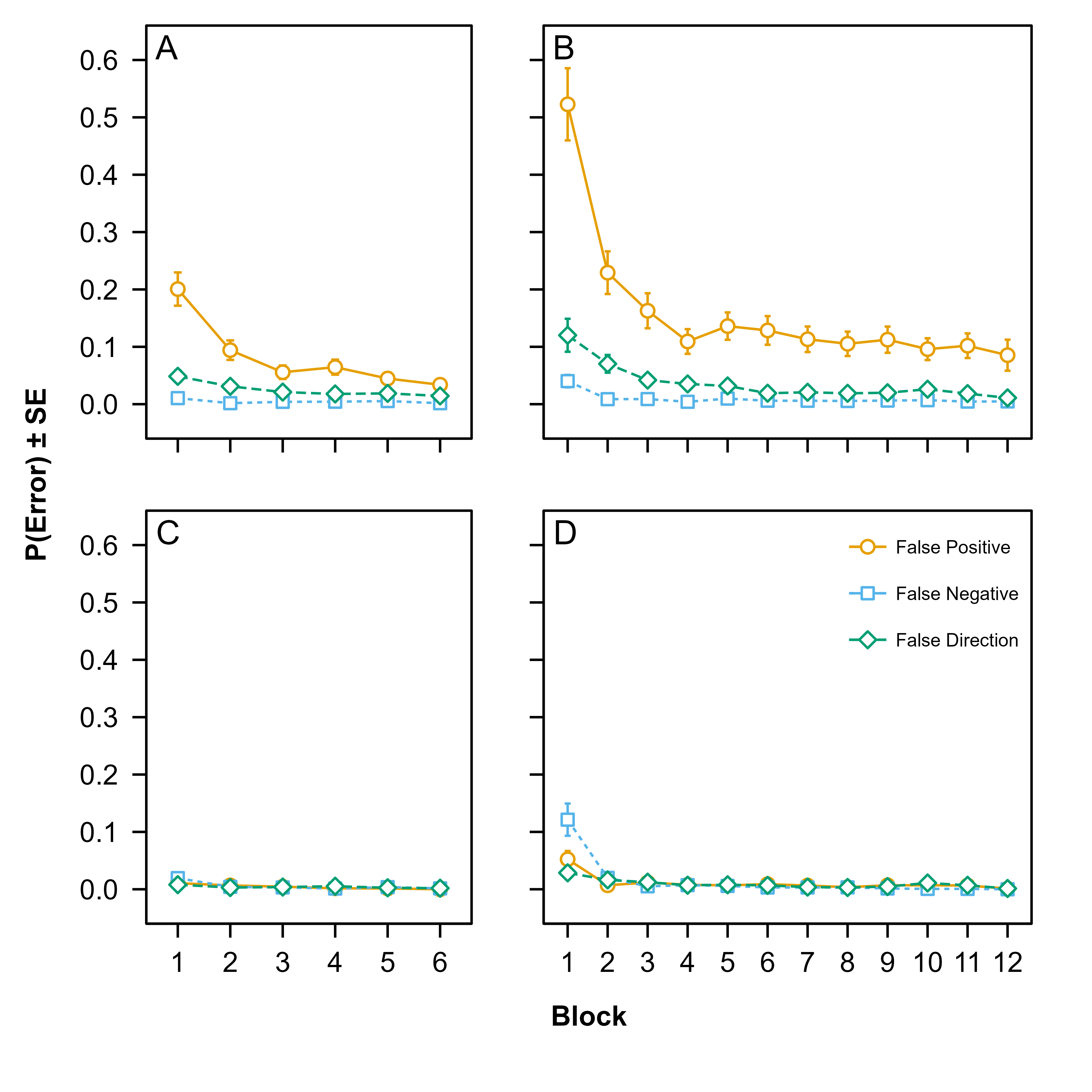


*Note*. Data points represent estimated marginal means (EMMs) predicted by generalized linear mixed models fit to the probability of different types of response error. Panels A and B display saccade error rates in Experiments 1 and 2, respectively, whereas Panels C and D display vocal error rates in Experiments 1 and 2. Three error types are distinguished: false-positive errors (saccades executed during single-vocal trials and vocal responses executed during single-saccade trials), false-negative errors (omitted saccades in single-saccade or dual-action trials and omitted vocal responses in single-vocal or dual-action trials), and directional errors (saccades made toward the incorrect location and vocal responses naming the incorrect direction). Probabilities are computed across all trials in which the respective error could occur. Error bars represent model-based standard errors of the EMMs.

## Saccade RTs

Table S2

Results of LMMs Fitted to Saccade RTs

| Experiment | Effect | $F$ | ${df}^{S}$ | ${df}_{\mathrm{res}}^{S}$ | $p$ |
| --- | --- | --- | --- | --- | --- |
| 1 | Block | 7.28 | 5 | 40.58 | < .001 |
| 1 | Trial Type | 0.75 | 1 | 26.03 | .395 |
| 1 | Block $\times$ Trial Type | 0.97 | 5 | 5720.02 | .432 |
| 2 | Block | 18.39 | 11 | 49.41 | < .001 |
| 2 | Response | 0.10 | 1 | 26.97 | .752 |
| 2 | Block $\times$ Trial Type | 3.20 | 11 | 11053.42 | < .001 |

*Note*. Fixed-effect tests from linear mixed models predicting saccade RTs (ms) as a function of time-on-task (Block) and Trial Type. *F* statistics are Type III tests of fixed effects. Degrees of freedom were estimated using the Satterthwaite approximation. Separate models were estimated for Experiment 1 and 2. All models included random intercepts for participants (with random slopes where justified).

Figure S2

Estimated Marginal Means of Saccade RTs


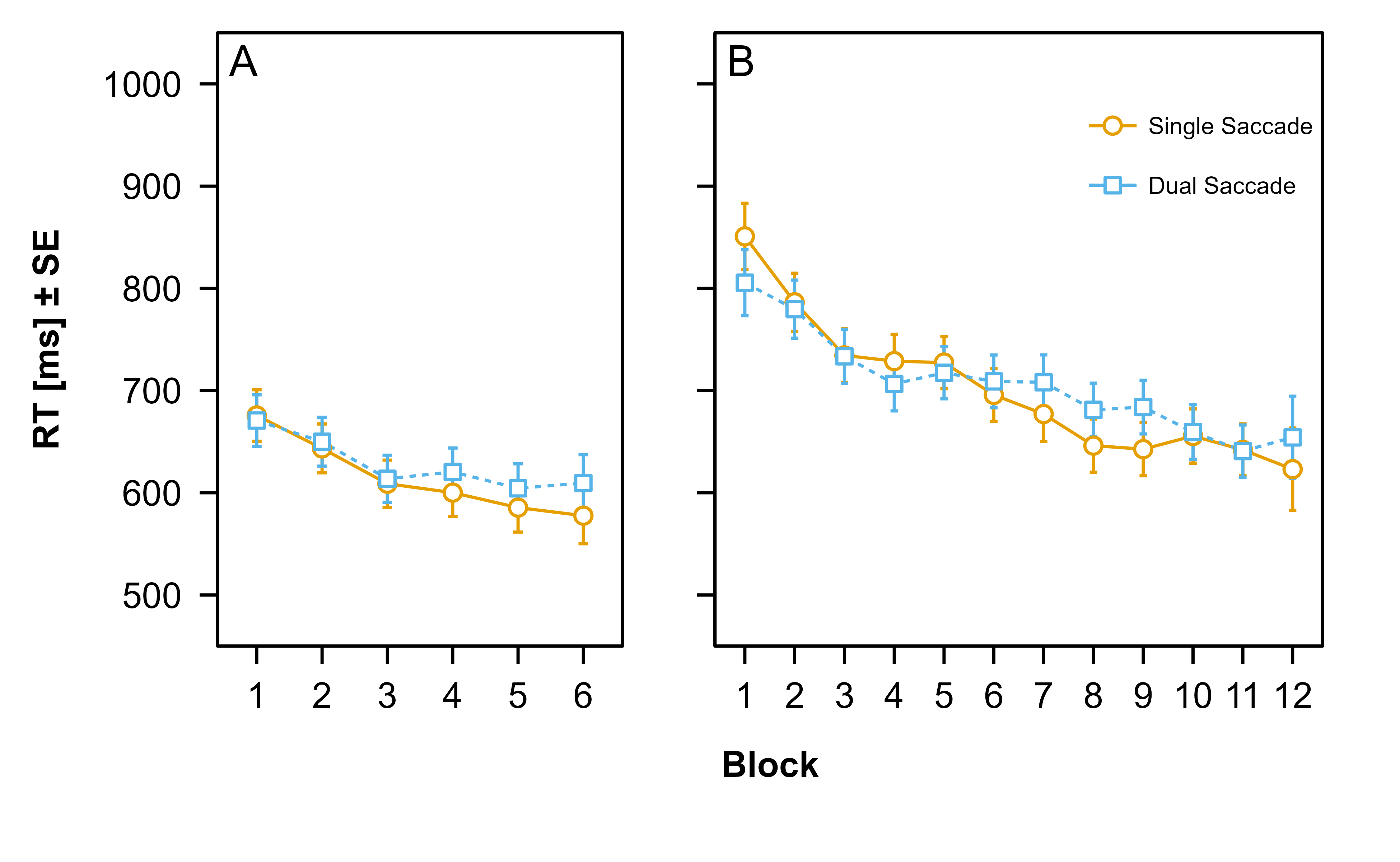


*Note*. Data points represent estimated marginal means (EMMs) predicted by the linear mixed models fit to saccade RTs in single-saccade and dual-action trials in Experiment 1 (A) and Experiment 2 (B), respectively. Error bars represent model-based standard errors of the EMMs.

## Inter-Response Intervals

Table S3

Results of LMMs Fitted to Inter-Response-Intervals

| Experiment | Effect | $F$ | ${df}^{S}$ | ${df}_{\mathrm{res}}^{S}$ | $p$ |
| --- | --- | --- | --- | --- | --- |
| 1 | Block | 0.60 | 5 | 35.39 | .700 |
| 2 | Block | 2.66 | 11 | 52.82 | .009 |

*Note*. Fixed-effect tests from linear mixed models predicting inter-response intervals (ms) as a function of time-on-task (Block). *F* statistics are Type III tests of fixed effects. Degrees of freedom were estimated using the Satterthwaite approximation. Separate models were estimated for Experiment 1 and 2. All models included random intercepts for participants (with random slopes where justified).

Figure S3

Estimated Marginal Means of Inter-Response-Intervals


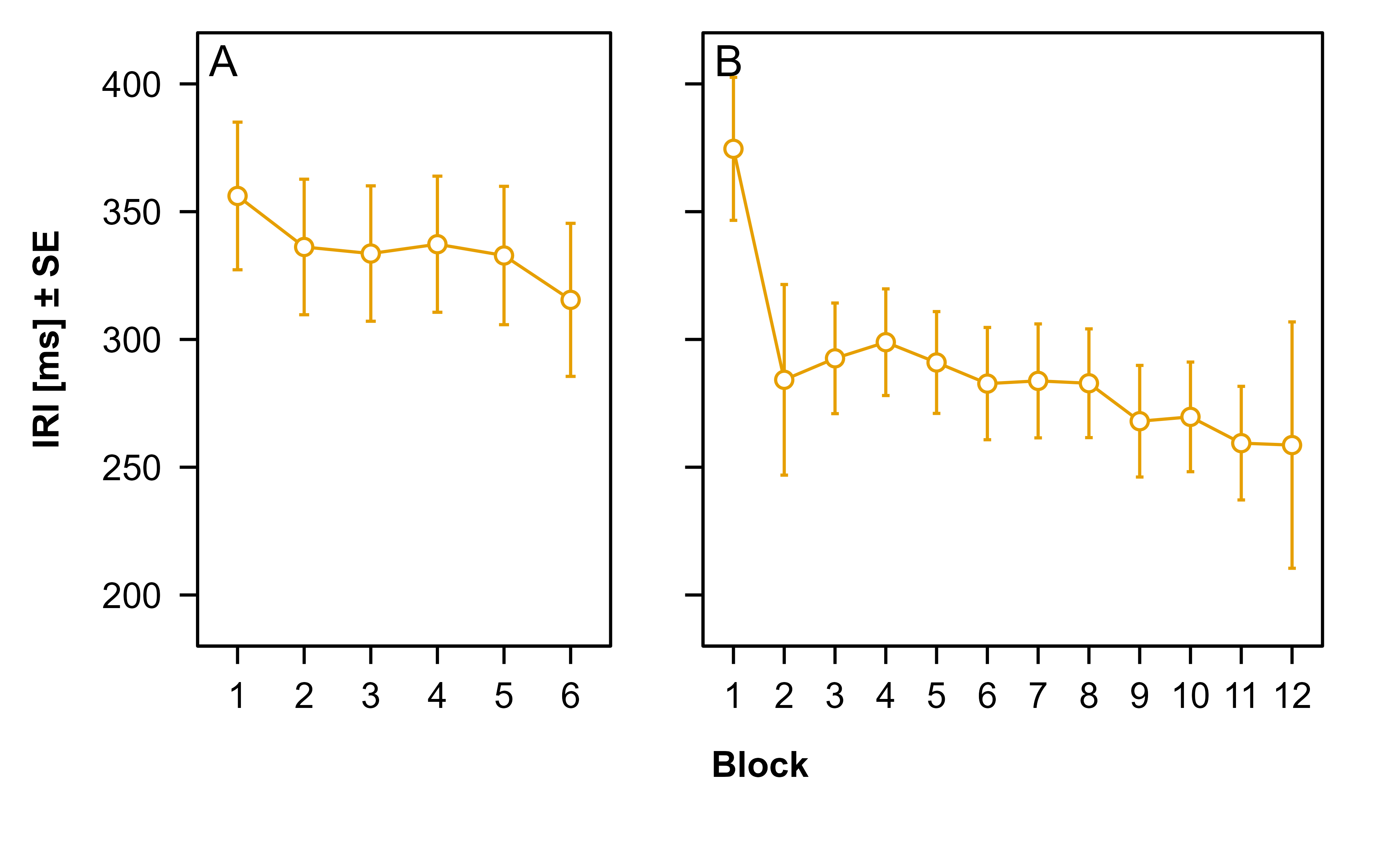


*Note*. Data points represent estimated marginal means (EMMs) predicted by the linear mixed models fit to inter-response intervals (IRIs) in correct dual-action trials in Experiment 1 (A) and Experiment 2 (B), respectively. Error bars represent model-based standard errors of the EMMs.
